# Theory and Applications of Covalent Docking in Drug Discovery: Merits and Pitfalls

**DOI:** 10.3390/molecules20021984

**Published:** 2015-01-27

**Authors:** Hezekiel Mathambo Kumalo, Soumendranath Bhakat, Mahmoud E. S. Soliman

**Affiliations:** Molecular Modelling and Drug Design Research Group, School of Health Sciences, University of KwaZulu-Natal, Westville, Durban 4000, South Africa; E-Mails: hezekiel1989@gmail.com (H.M.K.); bhakatsoumendranath@gmail.com (S.B.)

**Keywords:** covalent docking, covalent inhibition, covalent interactions, irreversible inhibition

## Abstract

The present art of drug discovery and design of new drugs is based on suicidal irreversible inhibitors. Covalent inhibition is the strategy that is used to achieve irreversible inhibition. Irreversible inhibitors interact with their targets in a time-dependent fashion, and the reaction proceeds to completion rather than to equilibrium. Covalent inhibitors possessed some significant advantages over non-covalent inhibitors such as covalent warheads can target rare, non-conserved residue of a particular target protein and thus led to development of highly selective inhibitors, covalent inhibitors can be effective in targeting proteins with shallow binding cleavage which will led to development of novel inhibitors with increased potency than non-covalent inhibitors. Several computational approaches have been developed to simulate covalent interactions; however, this is still a challenging area to explore. Covalent molecular docking has been recently implemented in the computer-aided drug design workflows to describe covalent interactions between inhibitors and biological targets. In this review we highlight: (i) covalent interactions in biomolecular systems; (ii) the mathematical framework of covalent molecular docking; (iii) implementation of covalent docking protocol in drug design workflows; (iv) applications covalent docking: case studies and (v) shortcomings and future perspectives of covalent docking. To the best of our knowledge; this review is the first account that highlights different aspects of covalent docking with its merits and pitfalls. We believe that the method and applications highlighted in this study will help future efforts towards the design of irreversible inhibitors.

## 1. Introduction

Computational and molecular modeling tools have become a close counterpart to experiment in the understanding of molecular aspects of biological systems [1,2,3,4]. The computational approaches like homology modeling, molecular docking and quantitative structure activity relationships (QSAR) and molecular dynamics (MD) are widely employed to discover the novel hits for various therapeutic targets. In a recent report, we have highlighted the interface between computational approaches and experiment as crucial tools in the drug discovery machinery [5]. With the prominent rising interest towards the design of covalent inhibitors, in this review we cover different aspects of covalent molecular docking as a tool that can be applied to understand covalent interactions between inhibitors and their target proteins.

## 2. Covalent Interactions in Biological Systems

**Figure 1:**
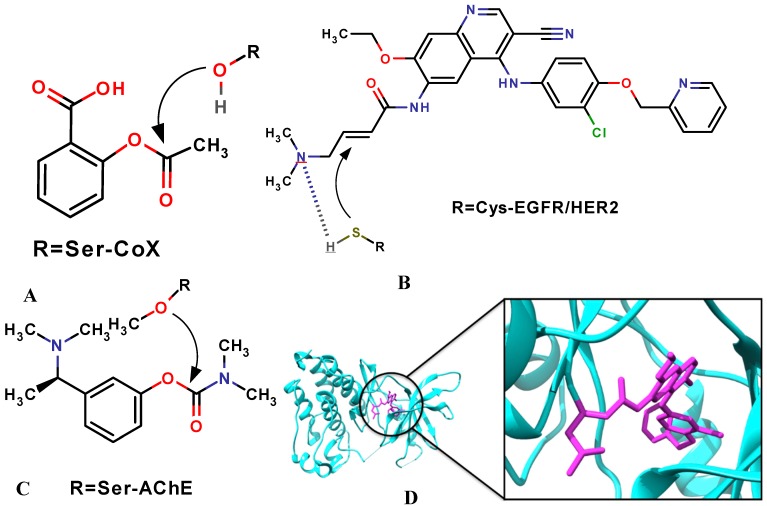
Examples of covalent inhibitors including their protein target(s) with active-site nucleophile: (**A**) aspirin; (**B**) neratinib; (**C**) rivastigmine; (**D**) Crystal structure of EGFR kinase domain in complex with neratinib, showing the covalent bond between the receptor and the ligand [27].

In recent literature, there has been a growing interest in the design of drugs forming a covalent bond with the target protein, with nearly 30% of the marketed drugs targeting enzymes known to act by covalent inhibition (Table 1) [6,7]. These types of inhibitors derive their activity from both non-covalent interactions and the formation of the covalent bond between the inhibitor and the target protein [8,9,10,11,12,13]. The covalent drugs typically have much stronger binding affinity, with the targets because of the covalent linkage formed between the ligand (electrophilic) and the target (nucleophilic), hence stronger potency while maintaining a pharmaceutically favoured small molecule size. Covalent interaction with the target protein has the benefit of prolonged duration of the biological effect. However, these types of inhibitors tend to be associated with toxicity because of the difficulty of disassociation if off-target binding happens. Therefore, highly specified selectivity profiles of the covalent drugs are required. It is reported that approximately, 33% of the covalent drugs in the market are anti-infectives (most notably the β-lactam class of antibiotics), 20% treat cancer, 15% treat gastrointestinal disorders, and ~15% are used to treat central nervous system and cardiovascular indications [14]. The earliest example of a covalent drug is aspirin, which was first marketed over a century ago; aspirin covalently modifies cyclooxygenase by inducing the acetylation of a serine residue that is situated in the active site (Figure 1A) [15,16,17,18]. β-lactam antibiotics are other examples of a covalent drug which acylate the active site serine of penicillin-binding proteins (PBPs) and kill the bacteria by inhibiting the final step of cell wall biosynthesis. Tetrahydrolipstatin is another class of covalent inhibitors that inhibits fat absorption [19,20,21,22]. The reaction occurs between the β-lactone and the serine nucleophiles of the lipases to form stable ester bonds. Rivastigmine is a cholinergic agent for the treatment of mild to moderate dementia of the Alzheimer’s type (Figure 1C) [23]. It is reported that the catalytic serine nucleophile (Ser-200) is carbamylated, with the phenol-leaving group, which is retained in the active site. Neratinib (HKI-272) (Figure 1B, D) is a tyrosine kinase inhibitor under investigation for the treatment of breast cancer and other solid tumors [24,25,26]. It contains a 4-(dimethylamino) crotonamide Michael acceptor that forms a covalent bond with a conserved cysteine residue, Cys-773 in EGFR and Cys-805 in HER-2.

**Table 1:** List of FDA-approved drugs that form covalent interactions with targets.

Drug	Biological Target	Therapeutic Domain
Amoxicillin	PBP	Anti-infective
Cefaclor/Ceclor	PBP	Anti-infective
Ceftriaxone/Rocephin	PBP	Anti-infective
Cefuroxime axetil/ceftin	PBP	Anti-infective
Cephalexin/keflex	PBP	Anti-infective
D-cycloserine/seromycin	Alanine racemase	Anti-infective
Fosfomycin/monurol	UDP-N-acetylglucosamine-3-enolpyruvyl-transferase	Anti-infective
Isoniazid	Enol-acyl carrier protein reductase	Anti-infective
Meropenem	PBP	Anti-infective
Omnicef	PBP	Anti-infective
Penicillin V	PBP	Anti-infective
Azacytidine	Methyltranferase	Cancer
Bortezomib	Protesome	Cancer
Decitabine/azadC	Methyltranferase	Cancer
Dutasteride/avodart	5-α-Reductase	Cancer
Exemestane/Aromasin	Aromatase	Cardio-vascular
Floxuridine	Thymidylate synthase	Cardio-vascular
Gemcitabine/gemzar	Ribonucleoside reductatase	Cardio-vascular
Proscar/finasteride	5-α-Reductase	Cardio-vascular
Rasagiline	MAO-B	Parkinson’s disease
Selegiline	MAO-B	Parkinson’s disease
Warfarin	Vitamin K reductase	Cardio-vascular
Vigabatrin/sabril	GABA-Aminotransferase	Anti-epileptic
Nexium/esomeprazole	H+/K+ ATPase	Gastro-intestinal
Orlistat/	Lipase	Gastro-intestinal
Prevacid/lansoprazole	H+/K+ATPase	Gastro- intestinal
Prilosec/omeprazole	H+/K+ATPase	Gastro-intestinal
Protonix/pantoprazole	H+/K+ATPase	Gastro-intestinal
Aciphex/rabeprazol	H+/K+ATPase	Gastro-intestinal
Aspirin	Cyclooxygenase	Inflammation
Disulfiram/antabuse	Aldehyde dehydrogenase	Chronic alcoholism
Eflornithine	Ornithine decarboxylase	Hirsutism
Propylthiouracil/procasil	Thyroxine-5-deiodinase	Hyperthyroidism
Saxagliptin/Onglyza	DPP-IV	Anti-diabetic drug
Vildagliptin/Eugreas	DPP-IV	Anti-diabetic drug
Phenoxy-benzamine hydrochloride	α-Adrenoceptor	Cardio-vascular
mercaptopurine/purinthol	Purine-nucleotide synthesis	Cancer
Carbidopa/lodosyn	DOPA decarboxylase	CNS

## 3. Molecular Docking: Non-Covalent and Covalent Docking

Molecular docking is a computational procedure performed on structure-based rational drug design to identify correct conformations of small molecule ligands and also to estimate the strength of the protein-ligand interaction, usually one receptor and one ligand [28,29,30]. The most common docking programs and software include Autodock [31], Autodock Vina [32], GOLD [33] and FlexX [34]. Yet, these and many other methods similar to them mainly focus on the docking between the two molecules through non-covalent interactions (van der Waals interaction, the electrostatics interaction and hydrogen bonding), or using other empirical or knowledge-based scoring functions to characterize these non-covalent interactions [34]. However, not all drugs bind non-covalently to the active site; there are other categories of drugs, namely the covalent drugs [14].

Docking of ligands that are bound to a receptor through non-covalent interactions is relatively conventional nowadays. The majority of docking methods development research has been focused on the effective prediction of the binding modes of non-covalent inhibitors [35,36,37,38,39,40]. However, docking ligands that bind covalently to the receptor has been complicated, because of the reaction between the ligand and the receptor that needs to be taken into consideration [15].

### 3.1. Covalent Docking: Theoretical Background

**Figure 2:**
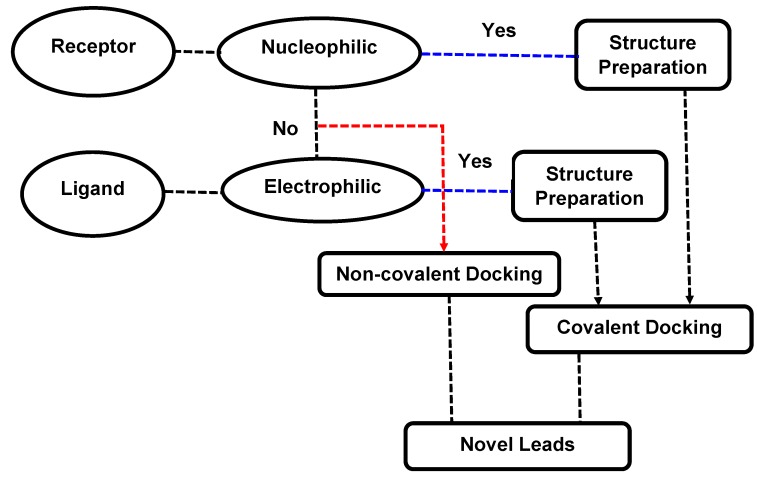
General scheme decribing the workflow of covalent docking in drug discovery.

Different routines have been developed to perform covalent docking of the inhibitors to the target proteins. However, most covalent docking softwares are only successful in predicting the binding energy between a nucleophilic receptor and electrophilic ligand (Figure 2).

One common routine is the “link atom” approach. In this approach the program defines a “link atom” both in the ligand and in the protein. This forces the ligand link atom to occupy the same steric volume as the protein link atom to mimic the covalent binding event. This approach is implemented in molecular docking software called Gold [41]. Autodock [31], another widely used molecular docking software, applies two methods to covalently dock inhibitors to receptor: a “grid-based method” and a “modification of the flexible side chain” approach. In flexible side chain, the covalent bound ligand and the protein attachment are treated as a single flexible side chain and sampled as part of the receptor. The grid-based method, utilizes a Gaussian biasing function with is centered on the protein attachment atom and also the grid-based energy to bias the covalent bonding ligand pose. However, the application of a covalent docking feature requires manual definition of the reactive atoms and reaction type as well as manual preparation of the ligand and protein structure files, leading to difficulties in up-scaling the process for screening purposes. Another program called CovalentDock has addressed this problem by automatic preparation of ligand files but is limited in reactions [42]. In covalent binding the ligand binds first through non-covalently interaction with the protein in a pose that benefits the reaction, and then followed by the reaction between the elements to form a covalent. Thus, in CovalentDock the interactions between the ligand and its receptor are modelled the same as in conventional molecular dockings through non-covalent interactions with additional energy contribution from covalent linkage formation is estimated by a newly formulated model (Equation (1)):

(1) $$E\left\{ \begin{array}{l} D\left(e^{-2\alpha \left(r-r_{0}\right)}-2e^{-\alpha (r-r_{0})}\right)-T\Delta S+C\begin{array}{c} \begin{array}{cc} \begin{array}{cc} \begin{array}{cc} & r\leq r_{m} \end{array} & \end{array} & \end{array}\\ r>r_{m} \end{array}\\ 0, \end{array}\right.$$

where, r_m_ is the maximum bond length without disassociation (which is defined as the distance when E = 0 where r > r_0_). α is a parameter controlling the well width, r is the bond length, and r_0_ is equilibrium bond length ΔS_est_ is the conformation entropy estimated by Gaussian, C is a correcting empirical constant. In generally the standard enthalpy change (Equation (2)) only provides estimation on the perfect bond length, of sometime in molecular docking, that length might not be perfectly optimal. Ouyang *et al*., formulated the Morse potential with parameters fitting to simulated result from Gaussian. The enthalpy change brought by covalent bond formation on sub-optimal bond length. The enthalpy change of a given covalent bonding pair given by the Morse potential is show in Equation (3):

(2) ΔG=ΔH−TΔS

(3) $$\Delta H=D\left(e^{-2\alpha \left(r-r_{0}\right)}-2e^{-\alpha (r-r_{0})}\right)$$

where, D is the dissociation energy, α is a parameter controlling the well width, r is the bond length, and r_0_ is equilibrium bond length. The parameterization of this equation is subjected to the specified type of covalent bonding pairs. This new approach was successfully tested with the pose prediction of 76 covalently bound complexes and a virtual screening study [42].

### 3.2. Implementation of Covalent Docking in Drug Discovery Workflows

**Figure 3:**
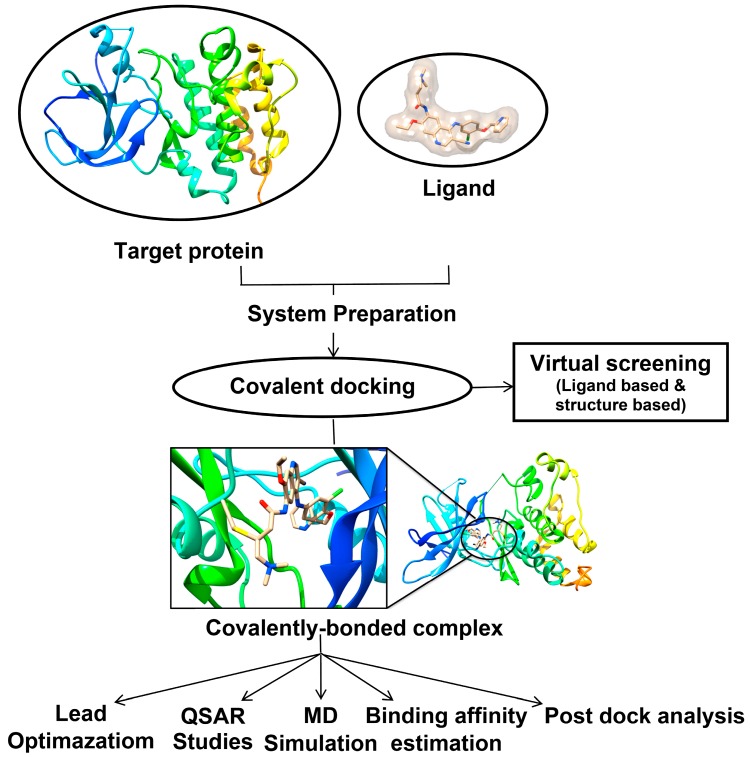
Implementation of covalent docking within computer-aided drug design approaches. In case of covalent docking target protein explored the interaction between nucleophilic target protein and electrophilic ligand.

Figure 3 highlights the different applications of covalent docking that could be implemented within drug discovery workflows. In the following section (Section 4) we provide an up-to-date literature survey on the different computer-aided drug design approaches that utilize covalent molecular docking as a tool to describe covalent enzyme inhibition.

## 4. Case Studies: Applications of Covalent Docking in Drug Design

Covalent ligands gained significant less attention in the traditional process of drug discovery due its off-target reactivity as well as toxicity profile. However the discovery of telaprevir and boceprevir, two FDA-approved covalent inhibitors targeting HCV, re-emphasized the focus on covalent inhibitors. Covalent inhibitors possessed some unique advantages e.g.,: (i) covalent warheads can target rare, non-conserved residue of a particular target protein and thus led to development of highly selective inhibitors; (ii) covalent inhibitors can be effective in targeting proteins with shallow binding cleavage which will led to development of novel inhibitors with increased potency than non-covalent inhibitors [43].

Katritch* et al*., applied covalent docking in conjunction with homology modeling to explore a detailed structural model of the ubiquitin-like poxvirus proteinase (ULP) I7L substrate-binding site (S2–S2'). The 3D model of the I7L ligand-binding site was then utilized to perform covalent docking and virtual screening of a comprehensive library of about 230,000 available ketone and aldehyde compounds to search for novel smallpox antiviral hits. Out of 456 predicted ligands, 97 inhibitors of I7L proteinase activity were confirmed to be active in biochemical assays (20% overall hit rate) [44].

**Figure 4:**
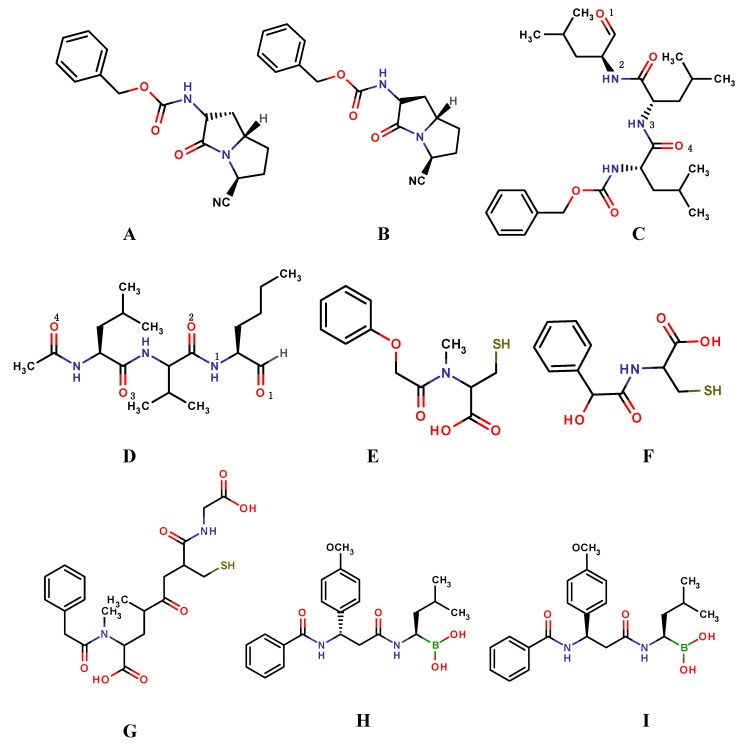
Covalent inhibitors, (**A**,**B**) bicyclic scaffolds; (**C**) MG132; (**D**) MG101; (**E**) NAC; (**F**) NMC; (**G**) GSH; (**H**,**I**) dipeptidyl boronic acid proteasome inhibitors.

In 2009, Lawandi* et al*., designed a class of covalent inhibitors from bicyclic scaffolds to study the optimal shape required for these small molecules to target propyl oligopeptidases (POP) to treat human brain disorders [45]. These structures bear nitrile functional groups that we previously predicted to be able to bind covalently to the catalytic serine of the enzyme. From the covalent docking study two compounds (Figure 4A,B) were selected for synthesis and subjected for biological assay. One compound was identified as a potent, highly selective, and cell-permeant POP inhibitor. Furthermore, the docking studies (using the FITTED docking engine and defaults parameters) also identified the configuration of the stereo-genic center at the ring junction as a limiting factor for optimal activity of which this can be used to develop second generation of potent inhibitors [45].

20S proteasome have an important role in the regulation of several important cellular processes, therefore is has been an attractive target in the field of anti-tumor research. Peptide aldehydes have been previously reported to inhibit the 20S proteasome activity by covalently binding to the active site of the β subunits.

Zhang* et al*., reported covalent docking (using GOLD version 4.0) in conjunction with molecular dynamics (MD) simulation (Amber Molecular Dynamics Package version 8.0) to explore the binding mode of peptide aldehyde inhibitors as anti-tumor drugs (Figure 4C,D). Form the covalently docking results, two conformations with the highest binding affinity (lowest docking energy) were selected subjected to molecular dynamics simulations. The binding mode analysis revealed that a space an aromatic group with a short linker at the position 4 site of the peptide aldehyde inhibitor (Figure 4C) would form favorable hydrophobic contacts with the neighboring subunit. A bulky substituent at the P2 position would also increase the binding stability by reducing water accessibility of the covalent bond. This study contributed to the understanding of the mechanism and structure-activity relationship of the peptide aldehyde inhibitors and may provide useful information for rational drug design [46].

Another study was conducted by Wang,* et al*., in which the authors described the application of covalent and 3D-QSAR Studies to explore the intermolecular interactions of isatin sulfonamide analogues as caspase-3 inhibitors. A series of 59 isatin sulfonamide analogues were docked into the binding site of the human caspase-3. The docking study provided an insight in the binding mode of the inhibitors. Furthermore, the use of a 3D-QSAR approach complements the docking analysis by providing a “custom” scoring function for the particular protein studied capable of predicting bioactivities for ligands similar to those found in the training sets. The study showed that structure-based design methods (such as docking) can cultivate the development of reliable QSAR models [47].

Recently, Juhl* et al*., applied substrate-imprinted docking, a technique that combines covalent docking, geometry optimization and geometric filter criteria to identify productive substrate poses, to model: (i) enantioselectivity of *Candida antarctica* lipase B and a W104A mutant; (ii) enantioselectivity and substrate specificity of *Candida rugosa* lipase and *Burkholderia cepacia* lipase; and (iii) substrate specificity of an acetyl- and a butyrylcholine esterase. The experimentally observed differences in selectivity and specificity of the enzymes were reproduced with an accuracy of 81%. The method was robust toward small differences in initial structures (different crystallization conditions or a co-crystallized ligand), although large displacements of catalytic residues often resulted in substrate poses that did not pass the geometric filter criteria [48].

In 2010, Chernorizov* et al*., reported an in *silico* screening study in order to identify a series of cysteine and glutathione derivatives as potential inhibitors of glyceraldehyde-3-phosphate dehydrogenase (GAPDH). This is a glycolytic enzyme reported to be directly involved in the apoptotic death of neurons in Parkinson’s disease. The compounds were theoretically capable of forming a disulphide bond with amino acid residue Cys14 (Figure 4E–G). Three compounds were discovered through covalent docking that showed high affinity to the NAD binding. The inhibitory effects of these compounds were tested on GAPDH from rabbit muscles using isothermal calorimetry and kinetic methods. From the biological assay two compounds that inhibited GAPDH. Cys149 is an important residue for interacting with Siah1. Therefore, the compounds were assumed to inhibit the formation of the proapoptotic complex GAPDH–Siah1 and therefore have potential effect against Parkinson’s disease [49].

Zhu* et al*., synthesized and modelled a series of novel dipeptidyl boronic acid proteasome inhibitors composed of β-amino acids. Docking results indicated that inhibitors termed “4q” and “4b” (Figure 4H,I) nearly interacted with 20S proteasome in a similar way as bortezomib. This indicated that they adopted a similar mode to inhibit the 20S proteasome as bortezomib [50].

**Figure 5:**
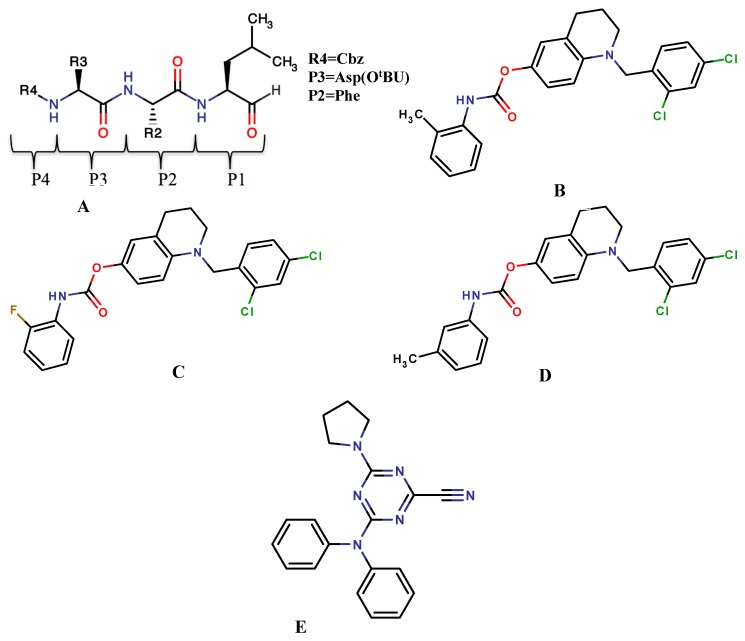
Covalent inhibitors: (**A**) proteasome inhibitor, (R = substituted groups and P = substituted amino acids); (**B**–**D**) carbamate derivatives; (**E**) CP243522.

In 2011, Ma* et al*., synthesized and covalently docked a new series of peptide aldehyde derivatives, which had a bulky P3 moiety aiming to increase the hydrophobic interactions (Figure 5A). Covalent docking was used to simulate the binding of the peptide aldehyde compounds with 20S, and the docking mode is similar to that of the observed crystal complex and that the P3-postion substitutes are crucial for inhibitor potency. The suggested binding mode provides a potential way to design more potent inhibitors of the 20S proteasome [51].

In 2012, Roy* et al*., reported the synthesis and pharmacological evaluation of a novel series of 16 carbamates. Among the 16 compounds, only three compounds (Figure 5B–D) exhibited promising* in vitro* AChE inhibitory activities comparable to the existing drug rivastigmine. Furthermore, the AChE–carbamate Michaelis complexes of these potent compounds including rivastigmine and ganstigmine were modeled by means of covalent docking and important structural factors governing the complex stability observed. Analyses of docking results revealed that rivastigmine and ganstigmine had a distinct orientation with respect to the active site architecture of TcAChE enzyme, even though they shared the same binding site. The interaction patterns of the two new potent carbamates (Figure 5B,D) were very similar to that of ganstigmine [52].

In 2013, Schröder* et al.* presented the implementation of a docking-based virtual screening workflow for the retrieval of covalent binders, human cathepsin K was utilized as a test case. A set of 44 candidate compounds with unknown activity on cathepsin K selected for biological. The most potent inhibitor, 4-(N-phenylanilino)-6-pyrrolidin-1-yl-1, 3, 5-triazine-2-carbonitrile (CP243522) (Figure 5E), showed a Ki of 21nM and was confirmed to have a covalent reversible mechanism of inhibition [53].

In 2014, Blake* et al*., reported the application of a unique hybrid ligand/structure-based virtual screening using covalent docking to search for irreversible protein splicing inhibitors as potential anti-TB drugs. The method was validated by means of MD simulation to ensure that docked complexes are stable and there were no possible docking artifacts [54]. Also in 2014, Dong* et al.*, showed that covalent docking can be a useful tool for substrate discovery, they investigated the accuracy of docking poses and substrate discovery in the GST superfamily, by docking 6738 potential ligands from the KEGG and MetaCyc compound libraries into 14 representative GST enzymes with known structures and substrates [55].

## 5. Software and Web Servers for Covalent Docking

To address the challenges of covalent docking, several popular non-covalent docking software’s implemented covalent docking protocols to understand covalent interaction between ligand and receptor. Besides standalone docking software, several research groups implemented covalent docking capability on cloud to perform covalent docking using web servers (Table 2). Implementation of covalent docking gets a huge boost with incorporation of covalent docking code in the widely used non-covalent docking server DOCK. Recent implementation of web based large scale virtual screening (CovalentDock Cloud) with covalent docking capability is a huge boost forward to develop potent covalent inhibitors targeting a variety of diseases using structure based drug design approach [56].

**Table 2:** List of the most popular software’s and websites utilized for covalent docking.

Standalone Software	Webservers
CovalentDock [42]	CovalentDock Cloud [42] http://docking.sce.ntu.edu.sg/
Gold [41]	Dockovalent (Covalent Docking Server) [57] http://covalent.docking.org/
CovDock-VS [58]	DockingServer [59] http://www.dockingserver.com/web/
Autodock [60]	
Glide [61]	
CovDock [62]	

## 6. Covalent Docking: Pitfalls and Future Prospectives

In the recent literature there has been a resurgence of covalent drugs, which has led to increasing interest in computational modeling methods such as covalent docking [14]. A major problem of covalent inhibitors is their off-target reactivity due to the presence of electrophilic reactive groups. To neutralize this safety concern the off-target reactivity can be minimized by developing inhibitors with a balance of covalent and non-covalent interactions which will led to a similar binding specificity comparable to the optimized non-covalent inhibitors. However covalent docking has inherited problems similar to those faced by non-covalent docking such as poor scoring functions, with entropy, speed and accuracy leading the list of problems. For example, two of the most popular molecular docking packages, Autodock [60] and GOLD [41], do support covalent docking feature, but they have a major restrictions in their functionality, accuracy, and usefulness. In addition the lack of automation in setting up the experiment has also hampered the use of covalent docking since the preparation steps for covalent docking requires much effort. CovalentDock [42] and CovDock [62] have addressed this problem by automatic preparation of ligand files however this is limited in reaction types and protein rigidity [42]. CovalentDock currently outperforms the default covalent docking method found in Autodock and GOLD in terms of better structural agreement of the results compared to the native structures in PDB, as well in virtual screening test to retrieve the true active controls from a large library of decoys. Even though CovalentDock produces good accuracy in binding mode prediction and has automatic detection of reactive atoms using SMARTS patterns, it does not facilitate structure-based virtual screening (SBVS) due to calculation length. However, Warshaviak* et al*., has addressed this issue by the development of CovDock-VS which has shown to be a straightforward and efficient method that can be applied successfully in screening campaigns for covalent inhibitors [58]. However there is a need to develop a better tuning of the energy estimation model as more knowledge of the structure and binding energy for covalent binding becomes available. Further research is needed to include the side-chain flexibility into CovalentDock-VS especially the flexibility on covalent-bond-forming residues, which is expected to enhance the performance of covalent docking [58].

Protein flexibility is also an issue for covalent docking even though; there have been numerous developments towards it. Abagyan and Totrov developed a 4D-docking protocol for Internal Coordinate Mechanics (ICM), where the receptor conformation is the fourth dimension [63,64,65]. In this protocol, multiple grids represent multiple receptor conformations and each is represented as a variable in the global optimization. This approach presented an increase in accuracy with no loss in effectiveness compared to single grid methods. AutoDock 4 fully models the flexibility of selected portions of the protein [31]. The side chains that are selected by the user are separated from the protein and treated explicitly during the simulation, allowing rotation around torsional degrees of freedom. The issues related to receptor flexibility have been extensively reviewed in literature [28,66,67].

Therefore, protein flexibility could be one of the major future directions in protein-ligand docking (both covalent and non-covalent) to improve this obstacle. They have not been much research conducted towards the field of covalent docking therefore; this leaves room for a breakthrough which more likely to come from better scientific understanding of protein–ligand interplay translated into better scoring.

## 7. Conclusions

Although covalent docking has been implemented in different drug discovery schemes and proved to be a useful tool to model covalent interactions between inhibitors and their biological targets, it is still an avenue for challenge and improvements. Some crucial aspects such as the lack of accuracy, speed, ligand sampling and protein flexibility should be revisited with more improved algorithms to overcome such shortcomings.

## List of Abbreviations

AChE: Acetylcholinesterase
CNS: Central nervous system
Cys: Cysteine
DPP-IV: Dipeptidyl peptidase 4
DOPA: 3, 4-Dihydroxyphenethylamine
EGFR: Epidermal growth factor receptor
GAPDH: Glyceraldehyde-3-phosphate dehydrogenase
KEGG: Kyoto Encyclopedia of Genes and Genomes
GABA: γ-Aminobutyric acid
H+/K+ ATPase: Hydrogen potassium ATPase
MAO-B: Monoamine oxidase B
MD: Molecular dynamics
PARP: Poly-(ADP-ribose)-polymerase
POP: Propyl oligopeptidases
PBP: Penicillin-binding protein
QSAR: Quantitative structure activity relationships
ULP: Ubiquitin-like poxvirus proteinase I7L
SBVS: Structure-based virtual screening
